# Infection of *Burkholderia cepacia* Induces Homeostatic Responses in the Host for Their Prolonged Survival: The Microarray Perspective

**DOI:** 10.1371/journal.pone.0077418

**Published:** 2013-10-07

**Authors:** Vanitha Mariappan, Kumutha Malar Vellasamy, Jaikumar Thimma, Onn Haji Hashim, Jamuna Vadivelu

**Affiliations:** 1 Department of Medical Microbiology, Faculty of Medicine, University of Malaya, Kuala Lumpur, Malaysia; 2 Department of Molecular Medicine, Faculty of Medicine, University of Malaya, Kuala Lumpur, Malaysia; 3 University of Malaya Centre for Proteomics Research (UMCPR), University of Malaya, Kuala Lumpur, Malaysia; University of Iowa Carver College of Medicine, United States of America

## Abstract

*Burkholderia cepacia* is an opportunistic human pathogen associated with life-threatening pulmonary infections in immunocompromised individuals. Pathogenesis of *B. cepacia* infection involves adherence, colonisation, invasion, survival and persistence in the host. In addition, *B. cepacia* are also known to secrete factors, which are associated with virulence in the pathogenesis of the infection. In this study, the host factor that may be the cause of the infection was elucidated in human epithelial cell line, A549, that was exposed to live *B. cepacia* (mid-log phase) and its secretory proteins (mid-log and early-stationary phases) using the Illumina Human Ref-8 microarray platform. The non-infection A549 cells were used as a control. Expression of the host genes that are related to apoptosis, inflammation and cell cycle as well as metabolic pathways were differentially regulated during the infection. Apoptosis of the host cells and secretion of pro-inflammatory cytokines were found to be inhibited by both live *B. cepacia* and its secretory proteins. In contrast, the host cell cycle and metabolic processes, particularly glycolysis/glycogenesis and fatty acid metabolism were transcriptionally up-regulated during the infection. Our microarray analysis provided preliminary insights into mechanisms of *B. cepacia* pathogenesis. The understanding of host response to an infection would provide novel therapeutic targets both for enhancing the host’s defences and repressing detrimental responses induced by the invading pathogen.

## Introduction

*Burkholderia cepacia* is naturally found in relatively high population in moist soil, in particular the rhizosphere of plants and the freshwater environments [1]. It is a non-pathogenic bacterium found in normal, healthy individuals. However, this organism can become an opportunistic human pathogen associated with life-threatening pulmonary infections that affect immunocompromised individuals, particularly patients with cystic fibrosis (CF) [2] and chronic granulomatous disease [3]. Pathogenesis of *B. cepacia* has been reported to be multi-factorial, associated with the ability of the bacterium to adhere, colonise, invade, replicate, survive and persist in the host cells, as well as to evade the host immune response [4,5]. These organisms are also able to secrete virulence proteins which aids in bacterial penetration into the epithelial barrier and gain access to the underlying tissue as well as the bloodstream. However, the virulence of *B. cepacia* infection is dependent on the nature of the bacterial strain in terms of types of virulence factors involved, the number of organisms in the initial exposure, the age of the bacteria and status of the host immune response.

How the lung epithelial cells responds towards *B. cepacia* infection is still undefined. In recent years, there have been numerous reports of gene expression analyses performed using the microarray platform to study the host’s response to acute bacterial infection in the modulation of different host genes in different target tissues [6,7]. To date, there has been no report on transcriptional changes induced by *B. cepacia* in the host. In the present study, we determined the factors involved in virulence of *B. cepacia* i.e., release of the extracellular enzymes and effect of the bacterial multiplicity of infection (MOI) at different stages of growth on invasion and intracellular survival. Investigation was also performed on the transcriptional changes in the host response towards exposure to *B. cepacia* live bacteria (mid-log phase) and secretory proteins (mid-log and stationary phases) that has previously been identified using 2-DE and MS analyses [5].

The aim of this study was to develop a comprehensive profile of the host transcriptional responses that occur in the lung epithelial cells during an infection with *B. cepacia* live bacteria or exposure to its secretory proteins. This improves our understanding of the immediate host responses at the early stage of *B. cepacia* infection.

## Materials and Methods

### Bacterial strain, growth and culture conditions

*B. cepacia* CQK, isolated from a non-CF patient at the University Malaya Medical Centre (UMMC), Kuala Lumpur, Malaysia, was cultured on nutrient agar. The bacterial culture was prepared using Luria-Bertani (LB) broth according to protocols described by Mariappan et al. (2011) [5]. Bacterial culture was sampled at every 2 hours interval (0 to 24 hours) and centrifuged at 20,000*g* for 40 mins at 4°C, after which the supernatant was collected and filtered through a 0.22 µm pore size membrane filter to remove residual bacteria [8].

### Exoenzyme profile of *B. cepacia* secretory proteins

The bacterial free culture supernatant was concentrated 50-fold using ultrafiltration employing 10 kDa centricon ultra-free centrifugal filter units (Millipore, USA). The 10kDa cut-off was selected based on data obtained from our previous studies [5]. Total protein concentrations were determined using Bradford method (1976) [9]; protease was assayed using azacoll according to Chavira et al. (1984) [10]; phospholipase C (PLC) was assayed using *p*-nitrophenyl phosphorylcholine (NPPC), ( [11]; peroxidase activity was assayed using o-dianisidine [12]; acid and alkaline phosphatase activities were assayed by measuring the release of p-nitrophenol from p-nitrophenol phosphate (p-NPP) at OD_410nm_ [13]; lipase activity was assayed using Tween 80 [14]. The quantification of isocitrate dehydrogenase (ICD) was performed according to Anderson et al. (1991) [15] to determine bacterial cell lysis. In all the experiments, fresh LB was used as control and the assays were performed in triplicates.

### Invasion and intracellular survival assays

Approximately 5 x 10^5^ A549 cells were seeded into each well of a 24-well tissue culture plate containing 1 ml of Roswell Park Memorial Institute (RPMI) growth medium containing 10% foetal calf serum and incubated at 37°C overnight. The invasion and intracellular survival assays were performed as described by Mariappan et al. (2011) [5]. In brief, the A549 cells were infected with *B. cepacia* grown to three different time-points of growth (mid-log, early stationary and stationary) at different MOI -1:10, 1:50 or 1:100 and were incubated for 1, 3, 6, 12, 18 and 24 hours respectively, at 37°C in 5% CO_2_ to allow bacterial invasion. For the intracellular survival assay, following three hours of invasion, the extracellular bacteria were killed using antibiotics (1 mg/ml ceftazidime and gentamicin, respectively) for 2 hours. The cells were further incubated in the antibiotic-free RPMI medium for 1, 3, 6, 12, 18 and 24 hours. Serial dilutions of the lysate was prepared and plated on nutrient agar to determine the bacterial counts [16]. Non-invasive *Escherichia coli* was used as a negative control. This experiment was performed in triplicates and the results were averaged.

### A549 epithelial cell viability test

Approximately 1×10^6^ A549 cells were seeded into each well of a 6-well tissue culture plate containing 1 ml of RPMI growth medium and incubated at 37°C in 5% CO2 until approximately 80% confluency. The confluent monolayer of A549 cells were exposed to *B. cepacia* live bacteria cultured to mid-log phase with ratio of 1:10 to 1:200 and the filter-sterilised mid-log and early-stationary phase secretory proteins at different concentrations ranging from 0-100 µg/ml, for 3 hours at 37°C in 5% CO_2_. Following 3 hours, 0.1% trypsin was added to detach the cells, after which the cell suspension was centrifuged at 300*g* for 5 minutes. The pelleted cells were then resuspended in 1 ml of phosphate buffered saline (PBS) and the number of viable cells was enumerated on a haemocytometer using the tryphan blue exclusion method.

### Gene expression analyses

Three biological replicates of total cellular RNA from A549 cells were extracted subsequent to 3 hours exposure of *B. cepacia* culture supernatant (mid-log (ML) and early-stationary (ES) phases) and live bacteria (LBC) using the Qiagen RNeasy Mini Kit with on column DNase treatment according to the manufacturer’s instructions (Qiagen, USA). The integrity of the extracted RNA was assessed using the Agilent 2100 Bioanalyser (Agilent Technologies, USA) and a total of 500 ng RNA was amplified in a single-round of *in vitro* transcription amplification that allowed incorporation of biotin-labelled nucleotides using the Illumina TotalPrep RNA Amplification Kit according to the manufacturer’s instructions (Ambion, USA). Microarray experiments were performed using the Illumina HumanRef-8 BeadChip, (containing 24,526 distinct genes) according to the instructions provided by the manufacturer (Illumina, USA).

### Microarray data analysis

The data was analysed using two different softwares; Illumina’s BeadStudio version 1.0 (Illumina, USA) and GeneSpring GX version 10 (Agilent, California, USA) software. In brief, the BeadStudio was used to generate signal intensity values from the scans, followed by standard normalisation using the GeneSpring (substitution of 0.01 to expression values <0.01, per chip normalisation to 50th percentile, per gene normalisation to median). The normalised data were grouped on the basis of the experimental conditions (treated and non-treated) and filtered using the Scatter Plot. A Venn diagram was generated from the one-way ANOVA results in order to compare the number of genes significantly changed in each set (*p* value of ≤ 0.05 and an absolute change greater than 2-fold for *B. cepacia* infected cells relative to the un-infected control cells). The pathways of the differentially expressed genes was analysed using the Kyoto Encyclopaedia of Genes and Genomes (KEGG) mapper database (http://www.genome.jp/kegg/). The web-based software GOTerm Finder (http://go.princeton.edu/cgi-bin/GOTermFinder) and GeneTrail (http://genetrail.bioinf.uni-sb.de/) were used to analyse significant pathways. Selected data were organised by a hierarchical clustering with the web-based software Cluster 3.0. The clustering algorithm is based on an uncentred correlation metric, with average linkage clustering and visualised using Java Treeview V1.1.3.

### Quantitative real-time PCR (qRT-PCR)

qRT-PCR was performed in the IQ5 real-time PCR (BioRad Laboratories, USA), to verify and quantify the expression of *VAM 8, RGS2, IL-1β, NFKB1A, AKR1B10, PADI2, TNF* and *LTB*. All primers used for the targeted genes including *β-actin* and *GAPDH* were obtained from PrimerBank (http://pga.mgh.harvard.edu/primerbank/) (Table S1). All the primers were synthesised at Nano Life Quest Laboratories (Malaysia). Briefly, 25 µl reactions were performed using the iScript™One-Step RT-PCR kit with SYBR green according to the manufacturer’s instruction (BioRad Laboratories, USA), primers at a final concentration of 1 µM and a data acquisition temperature of 56°C. In order to control for variation in RNA concentration, cycle threshold (Ct) values were normalised to *β-actin* and *GAPDH* that does not change with infection. The IQ5 real-time PCR software (Biorad, California, USA) was used to generate the quality control of the replicates, data extraction and initial analysis with a manual threshold of 0.6 and an auto baseline applied in order to obtain the threshold cycle (Ct) value for each measurement taken.

## Results

### Determination of the optimal parameters for host interaction

**Figure 1:**
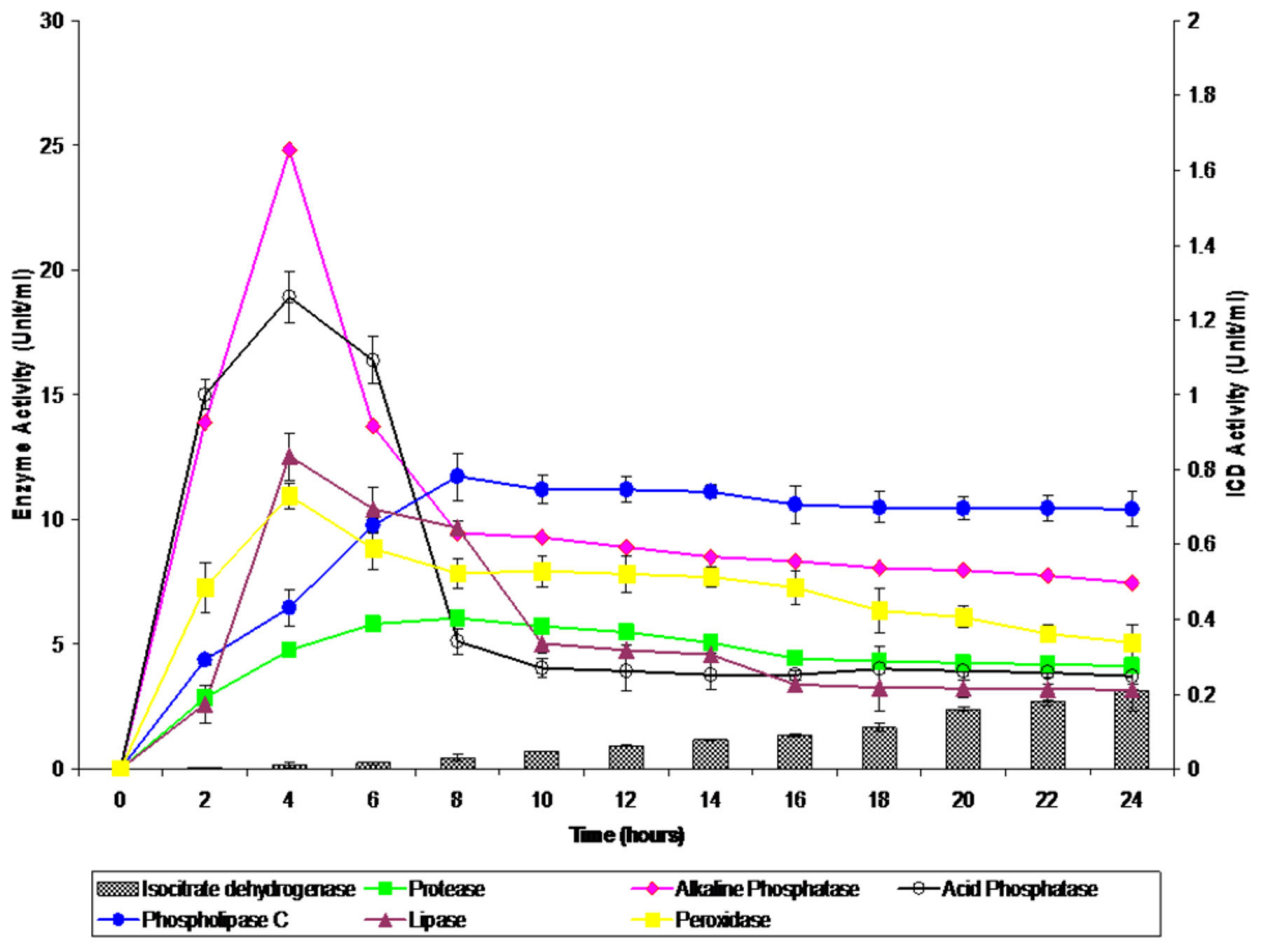
Exoenzyme profile of isocitrate dehydrogenase, lipase, phospholipase C, peroxidase, protease, acid phosphatase and alkaline phosphatase from *B. cepacia* secretory proteins at different phases of growth. The error bars indicate the standard deviation of triplicate values.

The secretion profiles of protease, peroxidase, lipase, PLC and acid and alkaline phosphatases were different for each of the enzymes studied at the different time points of bacterial culture (Figure 1). An intracellular enzyme assay, which detects ICD activity as a result of bacterial lysis was performed and insignificant levels of activity (0-0.21 Units/ml) were detected. Maximum enzyme peak activities were observed at 4 hours of growth (mid-log phase) for peroxidase, lipase, acid phosphatase and alkaline phosphatase with 10.96 Units/ml, 12.53 Units/ml, 18.92 Units/ml and 24.81 Units/ml, respectively. In contrast, the activities of protease and PLC increased to their highest levels at 8 hrs of growth (early-stationary phase) and with activities of 6.06 Units/ml and 11.73 Units/ml, respectively.

The ability of *B. cepacia* CQK strain to invade the epithelial cells was measured and compared at three different phases of bacterial growth (Table 1). In general, the trend of the invasion was similar for all the phases of growth at MOI 1:10 and 1:50. However, the invasion capacity at MOI 1:50 were comparatively higher than MOI 1:10 at all times of exposure and growth phases. The highest percentage of invasion (2.11%) was observed at MOI 1:50, stationary phase, 24 hours post-infection. At MOI 1:100, the percentage of invasion was moderately higher than MOI 1:50 at 3-12 hours post-infection at all phases of growth. However, the rate of invasion was found to decrease distinctly following longer post-infection time at the respective growth phases.

**Table 1:** Mean percentage of invasion (%) with standard deviation MOI 1:10, 1:50 and 1:100.

Time (Hours)	Mean percentage of invasion (%) - MOI 1:10			Mean percentage of invasion (%) - MOI 1:50			Mean percentage of invasion (%) - MOI 1:100		
	Mid-log	Early-stationary	Stationary	Mid-log	Early-stationary	Stationary	Mid-log	Early-stationary	Stationary
3	0.0044 ± 0.0016	0.0088 ± 0.0032	0.0111 ± 0.0042	0.0089 ±0.0016	0.0155 ± 0.0016	0.0177 ± 0.0016	0.0144 ± 0.0031	0.0211 ± 0.0016	0.0244 ± 0.042
6	0.0111 ± 0.0016	0.0177 ± 0.0016	0.0222 ± 0.0642	0.0222 ± 0.0016	0.0289 ± 0.0042	0.0355 ± 0.0016	0.0277 ± 0.0042	0.0355 ± 0.0016	0.0389 ± 0.0016
12	0.0333±0	0.0555 ± 0.0157	0.1111 ± 0.0157	0.0777 ± 0.0157	0.1666 ± 0.0272	0.2222 ± 0.0314	0.1333 ± 0.0272	0.2 ± 0.0272	0.2555 ± 0.0157
18	0.2222 ± 0.0157	0.2666 ± 0.0272	0.3±0	0.2666 ± 0.0272	0.3555 ± 0.0314	0.4111 ± 0.0157	0.111 ± 0.1571	0.1777 ± 0.0314	0.2222 ± 0.0157
24	0.3333±0	0.4222 ± 0.1571	0.4777 ± 0.1572	0.7777 ± 0.1572	1.6666±0	2.1111 ± 0.1571	0.088 ± 0.1571	0.1111 ± 0.1571	0.1666 ± 0.2722

**Figure 2:**
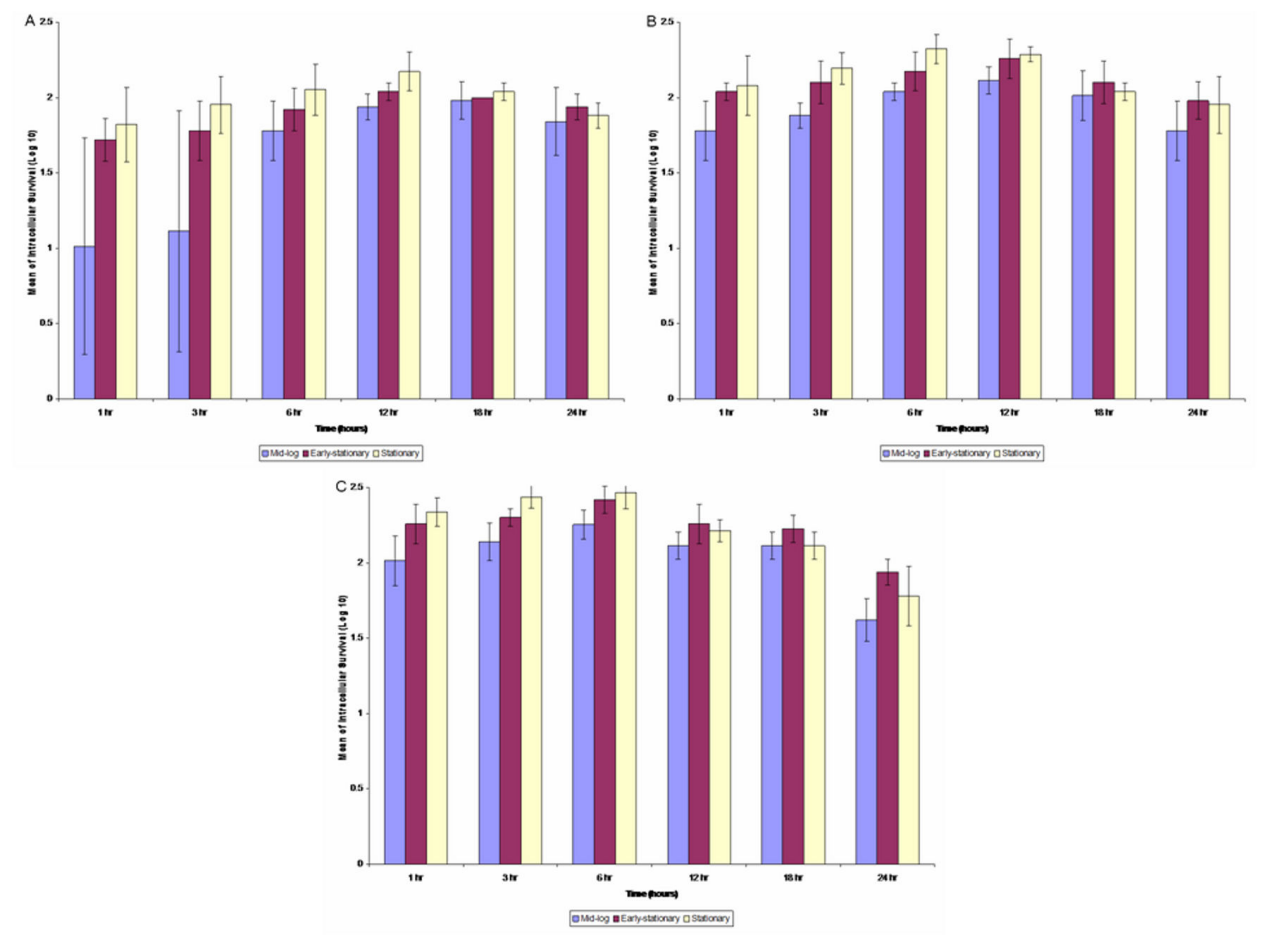
The intracellular survival assay of *B. cepacia* (mid-logarithmic, early-stationary and stationary phases of growth) after 3 hours of post-infection with MOI of (panel A) 1:10, (panel B) 1:50 and (panel C) 1:100 at different phases of growth. The error bars indicate the standard deviation of triplicate values.

In general, the intracellular survival profiles were similar at all the MOIs (Figure 2). The intracellular survival of *B. cepacia* at MOI 1:50 showed slow replication of *B. cepacia* in the epithelial cells from the first hour to 12 hours of incubation. A similar pattern was also observed with the intracellular survival of *B. cepacia* at MOI of 1:100. The number of bacteria replicated intracellularly peaked following six hours of incubation at all three phases. Nevertheless, it was noticeable that the number of cells living intracellularly decreased at longer time of replication (18 and 24 hours) at all the MOIs, suggesting that intracellular survival in the epithelial cells was saturable.

### Determination of epithelial cells viability upon infection of *B. cepacia*

**Figure 3:**
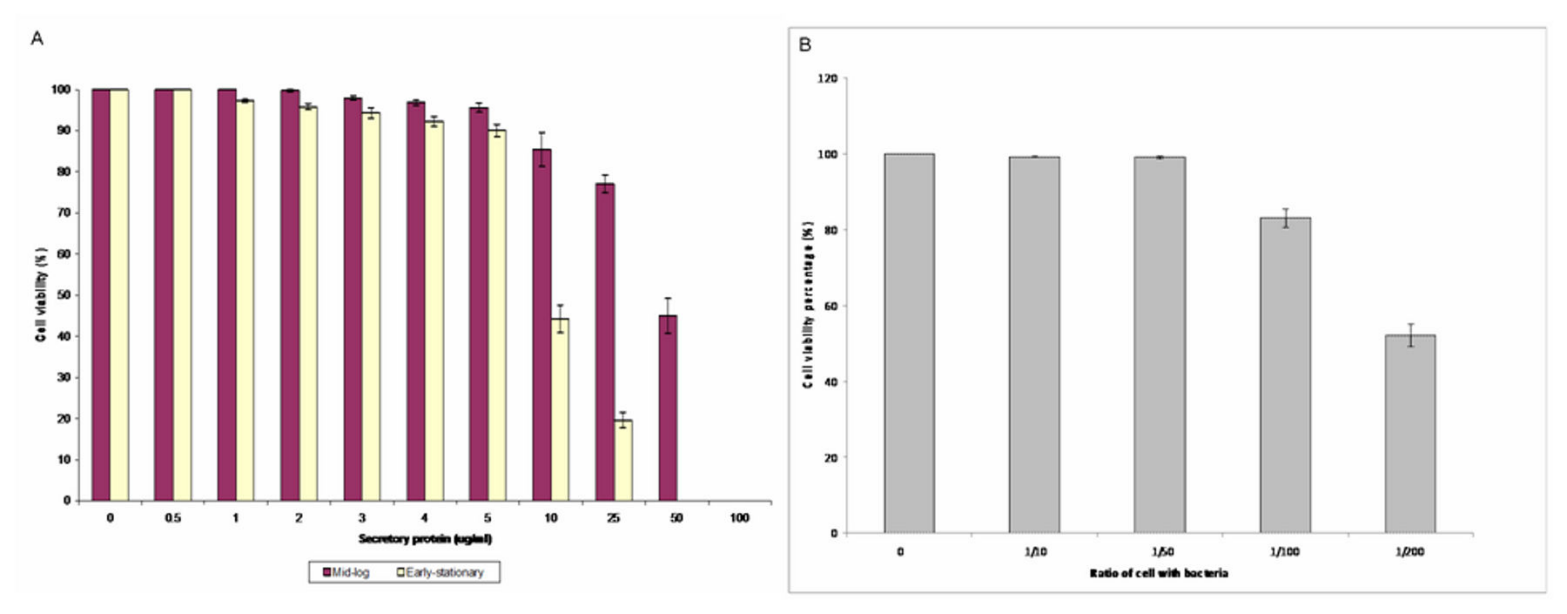
Percentage of A549 lung epithelial cells exposed to (panel A) secretory proteins (mid-logarithmic and early-stationary phases) ranging from 0 µg/ml-100 µg/ml and (panel B) live *B. cepacia* (mid- logarithmic phase) for three hours from 1:10 -1:200 using Trypan blue solution. The error bars indicate the standard deviation of triplicate values. The red box indicates the concentration choosen for microarray analysis.

The reduction in the percentage of the A549 epithelial cell viability was demonstrated using trypan blue (Figure 3, panels A and B). The cell survival was 100% at a concentration of 0.5 µg/ml culture supernatant harvested at the mid-log and early-stationary phases and decreased gradually as the concentration increased from 1-5 µg/ml. Using 50 µg/ml of the mid-log phase culture supernatant, viability of the host cells were found to be reduced to 55%. Conversely, none of the host cells survived when they were exposed to 50 µg/ml of the early-stationary phase culture supernatant (Figure 3, panel A). In addition, exposure of live *B. cepacia* to the A549 cells showed 99.2% and 99.05% of cell survival at MOI ratios of 1:10 and 1:50, respectively. However, the percentage of cell survival reduced to 83.02% at an MOI ratio of 1:100. Only 52.07% of the A549 cells were found to further survive at an MOI ratio of 1:200 (Figure 3, panel B).

### Host transcriptional responses to early exposure of *B. cepacia* live bacteria and the culture supernatant

**Figure 4:**
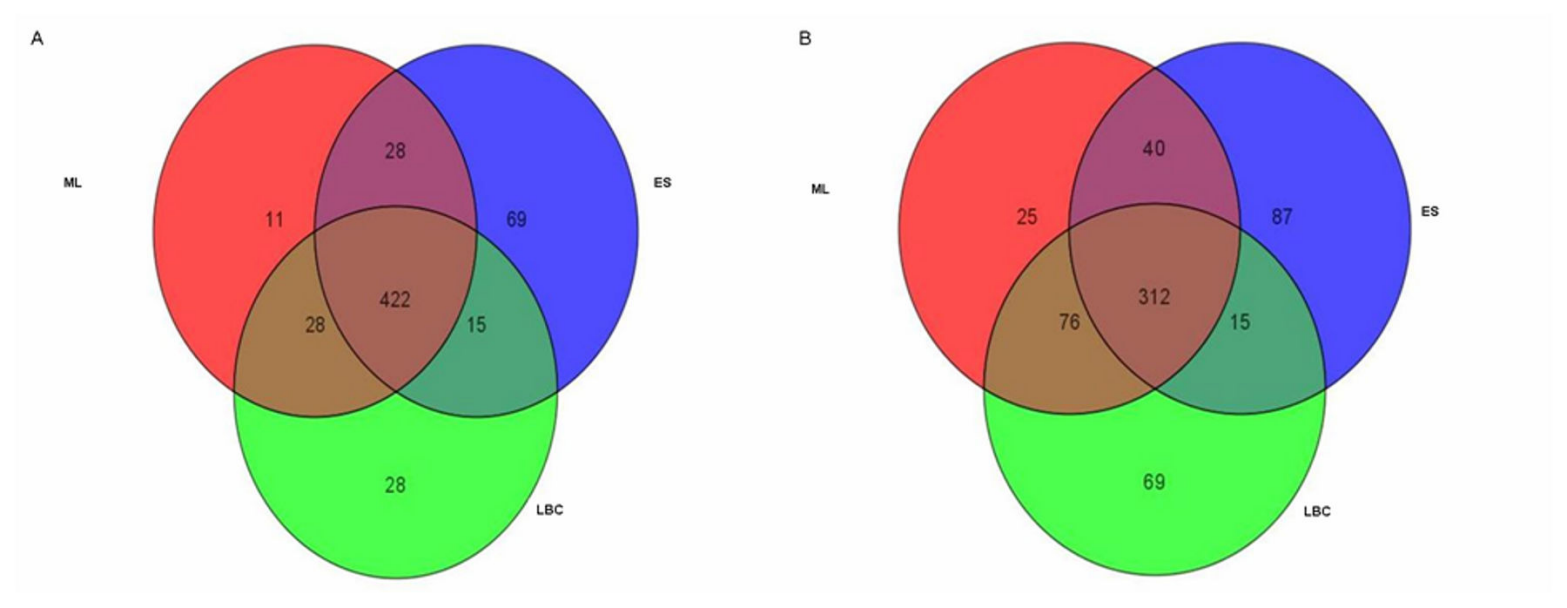
Venn diagrams showing the number of genes that were up-regulated (panel A) and down-regulated (panel B) with fold changes of ≥2.0-fold.

In this study, a total 20,496 of 24,526 genes, (83.57%) were filtered with cut-off values equivalent to Present and Marginal. Using the one-way ANOVA (asymptotic computed *p*-value <0.01) and Benjamini Hochberg (multiple testing corrections), 9,029 of 20,496 genes (44.02%) were identified as significantly expressed. Additionally, based on the results obtained from the fold change analysis, Venn diagrams were generated to determine common genes of the host that were differentially expressed (Figure 4, panels A and B). From the experiments, 422 genes were identified to be commonly up-regulated under all three experimental conditions (LBC, ML and ES). Conversely, only 312 genes were found to be commonly down-regulated among LBC, ML and ES. Scatter plots were also used to observe the relative distribution of genes that were significantly differentiated between each treated group and the control group.

**Figure 5:**
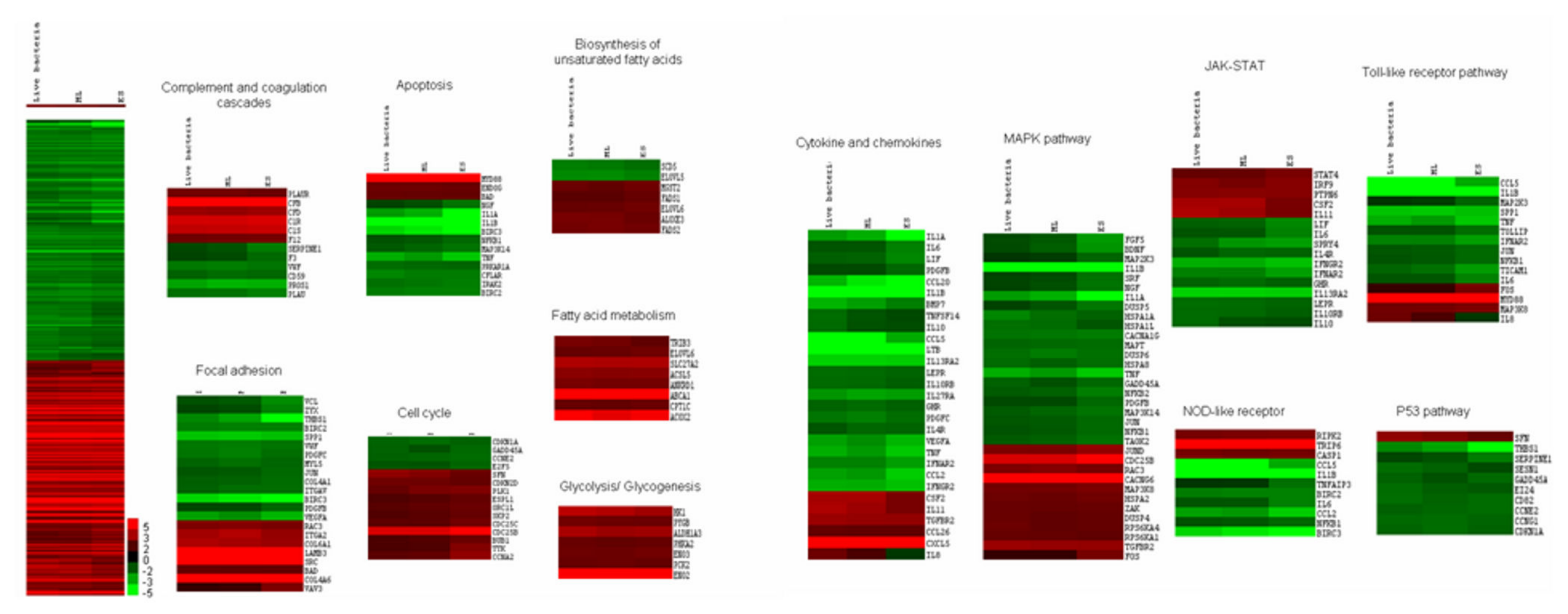
Heat map analysis.

Heat map analysis was performed and the significantly regulated genes (*p*-value<0.05) were classified according to the pathways (GeneTrail) and fold-change relative to the control and presented as hierarchical clustering of expression profiles based on functional categories (Figure 5). Several host immune regulatory pathways, cellular processes, metabolic pathways, regulation of cell cycle, apoptosis, inflammatory pathways that include numerous cytokines and chemokines were found to be differentially regulated upon exposure to live *B. cepacia* and the secretory proteins.

#### Activation of the host metabolic pathways

Expression of genes involved in the various metabolic pathways was significantly up-regulated under all the three conditions tested. The xenobiotics by cytochrome p450 metabolism and nicotinate and nicotinamide metabolism pathways were found to be enhanced in all the three conditions tested. However, glycolysis/glycogenesis and the arachidonic acid metabolism pathway were increased only in the LBC and ML conditions.

The majority of the genes identified were found to be involved in energy production, lipid metabolic processes, oxidation-reduction, cofactors and electron carrier. Glycolytic/ glycogenesis enzymes were significantly up-regulated including *PYGB, ENO* 2 and 3*, PHKA2, PCK2, ALDH1A3* and *HK1*, which are the key enzymes involved in the conversion of glucose-6-phosphate to pyruvate. In addition, genes encoding enzymes involved in the metabolism of fatty acids and biosysnthesis of unsaturated fatty acids were also up-regulated. Interestingly, genes associated with the metabolism of xenobiotics by cytochrome P450 (drug metabolism) were up-regulated in all the three conditions. This involves oxidases or detoxification of poisonous compounds i.e., *CYP* family, *MGST2*, *GSTO2* and *AKR1C2*.

#### Suppression of apoptosis and cell death programmes

Cell death related gene sets namely; apoptosis and NOD-like receptors pathway were down-regulated in all the conditions tested. In addition, several NOD-like receptors i.e., *CCL* 2 and 5, *BIRC* 2 and 3, *IL-6* and *IL-1β* and *NFκB1* were generally down-regulated. However, the subfamily of inflammatory mediator *CASP1* was activated together with nucleotide-binding domain and *NLRP2* to trigger the pro-inflammatory caspases upon injury, toxins or invasion. The p53 pathway was down-regulated only under the LBC, where *SFN*, which is the p53-regulated inhibitor of the G2/M progression in cell cycle, was up-regulated. However, the transcriptional of *CDKN1A*, *SERPINE*, *THBS1*, *GADD45A* and *SESN1* were down-regulated in the p53 pathway which resulted in inactivation of apoptosis, genomic stability and inhibition of angiogenesis. The apotosis pathway demonstrated that pro-apoptotic interleukins (*IL-1α* and *IL-1β*) associated with *IRAK2*, which was able to induce the NFκB1, were down-regulated.

#### Regulation of the host cell cycle and survival

The GeneTrail analysis revealed that most of the genes involved in the cell cycle, VEGF (involved in angiogenesis) and PPAR (involved in lipid oxidation and cell proliferation) signalling pathways were up-regulated. Genes that promote cell growth or arrest, DNA replication or repair and mitosis in the cell cycle pathway i.e., *SFN*, *SKP2*, *CDKN2D*, *ESPL1*, *ORC1L*, *BUB1* and *TTK*, were up-regulated (mainly under ES condition). However, the transcript levels of genes involved in stressful growth arrest conditions and treatment with DNA damaging agents including *CDKN1A*, *GADD45A*, *CCNE2* and *E2F5* were down-regulated under the ML and ES conditions.

#### Alteration of inflammation and cytokine-cytokine receptor interaction

The majority of the inflammatory and signal transduction pathways i.e., MAPK signalling, JAK/STAT, TLR signalling, chemokines and interleukins were generally down-regulated in all the three conditions. Several genes encoding the MAPK signalling pathway proteins of the activator protein-1 (AP-1) complex i.e., *JUND*, which protect cells from p53-dependent senescence and apoptosis, were up-regulated. Interestingly, *MKP* (oxidative or heat stress and growth factors gene) was found to be down-regulated by secretory proteins of the ML phase but up-regulated using secretory proteins under the ES and LBC conditions. In addition, it also acts on damaged proteins, causing partial unfolding and possible aggregation which directly inhibits apoptosis.

Under the ES condition, *C-FOS* and *JUND* were found to be up-regulated, in addition to the down-regulation of *C-JUN*. *NGF, PDGFB, FGF*, *TGF* and *BDNF* were also down-regulated under the ES condition. Activation of *NFκB* appears as one of the key players of the gene regulation coordination. However, there was no significant difference in the expression under the ML and LBC conditions.

On the other hand, under the ES condition, the cytokine transcriptional regulation indicated down-regulation of *NFκB*. This is in agreement with the down-regulation of *TNFα, IL-1α* and *IL-1β*, which are known to be regulated by *NFκB*. There was up-regulation of interleukin genes (*IL-2, IL-3*, and *IL-6*) observed in all the conditions tested. Moreover, genes encoding *STAT 4*; the Src homology region 2 domain-containing phosphatase-1 (*SHP-1*; also known as tyrosine-protein phosphatase non-receptor type-6 (*PTPN6*)), protein inhibitor of activated *STAT-1* (*PIAS1*) and protein 48 (*P48*; interferon response factor (*IRF9*)) were also up-regulated under all the experimental conditions.

TLRs are innate signalling receptors and play an important role in the induction of various immune responses, particularly the inflammatory related genes which were significantly differentiated upon contact of the live *B. cepacia* or secretory proteins with the host cell. *MyD88* (an important regulator of the TLR signalling pathway) and *MAP3K8* were significantly up-regulated under all the three conditions tested. However, there is also an apparent decrease in some cytokine end products of the TLR signalling observed under the ES secretory proteins, but no significant difference in expression was observed under the LBC and ML conditions. The expression of the COX-2 isoform was induced after interaction with live *B. cepacia* and secretory proteins (ML and ES).

Genes of the chemokines C-X-C motif chemokine 5 (*CXCL5*) as well as *IL-11* of the host were up-regulated after interaction with live *B. cepacia* and secretory proteins (mid-log and early-stationary phases). However, *CCL26* and *IL-8* were found to be up-regulated only under the LBC condition. On the other hand, *CCL2, CCL5* and *CCL20* genes along with *IL-1β*, *IL-1α, IL11RA* and *IL27RA* were down-regulated under the ML and ES conditions. In addition, *IL10RB* was down-regulated under the LBC and ML conditions whereas *IL-6* and *LIF* were down-regulated under the ES condition. 

### Validation of the microarray data

**Figure 6:**
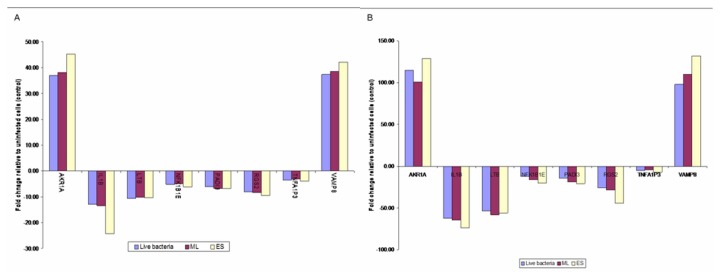
The relative relation between (A) microarray and (B) qRT-PCR assay.

The qRT-PCR assay was used for validation of genes involved in adhesion, cell growth, apoptosis, inflammation, and corresponding to cytokines, receptors, adapters, kinases, phosphatases, and transcription factors that were differentially expressed in the microarray analysis. The data obtained, together with those determined by the microarray experiments are shown in Figure 6 (panels A and B). The gene expressions obtained using the two techniques were comparable. However, differences were observed in the fold-change values whereby the fold-change detected in qRT-PCR assay was higher than the microarray.

## Discussion

In the present study, we have modeled an *in vitro* experiment to compare the host response between the actively dividing and adhering live *B. cepacia* or their secretory proteins. Previous studies on host-pathogen response of intracellular pathogens have focused primarily on a subset of genes from different pathways or limited gene expression patterns which is inadequate to dissect the host responses to an infection [7]. Our analyses clearly demonstrated that the pathogen had modulated the host metabolism, cell death, cell survival and innate immune system. To the best of our knowledge, the present study is the first to report the transcriptional host response of human airway epithelial cells after an interaction with live *B. cepacia* or with *B. cepacia* secretory proteins. This comparison provides a comprehensive genome wide view of the host transcriptional response and a deeper insight into the host response during pathogenesis of *B. cepacia*.

Various studies have demonstrated the enzyme activities of secreted proteins and their potential role in virulence of *B. cepacia* [14,17,18]. In this study, the secretion kinetics of six housekeeping exoenzymes of *B. cepacia* were selected based on their potential roles in the pathogenesis of *B. cepacia* [19]. The variation in the secretion kinetics of the different enzymes suggests differences in the enzymatic and pathogenic potential of the bacterium at different points of the growth cycle. In an *in vivo* condition, these enzymes may also be produced in large amounts at the logarithmic phase, in order for the bacteria to obtain nutrients for their multiplication as well as colonisation and subsequent invasion of the host [20].

In addition, invasion and intracellular survival are also important elements in the pathogenesis of *B. cepacia* whereby many studies have shown that *B. cepacia* were able to invade and survive within the epithelial cells *in vitro* and *in vivo* [4,21-25]. In the present study, the ability of *B. cepacia* to invade and multiply inside epithelial cells was measured based on the different pathogenic potentials in terms of profiles of exoenzyme activities. Our results suggest that the invasion efficiency of *B. cepacia* was directly related to the phases of growth and the MOIs. Collectively, these factors may contribute to the virulence potential of pathogenic *B. cepacia*. In order to survive and replicate efficiently in the host cells, it is necessary for the intracellular pathogens to adapt their metabolism to the available nutrients and physical conditions in the cells (mainly pH, oxygen availability and osmotic pressure), and also coordinate the metabolism with their life cycle.

The host-pathogen response may be reflected by the interaction of host cells with invading bacteria or their products, or to specific host defensive mechanisms. Upon exposure to live *B. cepacia* and its secretory proteins, genes associated with the host metabolic pathway were demonstrated to be up-regulated. This is compatible with the results shown by Moreilhon and co-workers (2005) [7], which demonstrated that the metabolic pathways were up-regulated in the human airway cells upon contact to live *Staphylococcus aureus* and its bacterial soluble factors. In our study, among the important metabolic pathways that were up-regulated was the xenobiotic metabolism-cytochrome p450 superfamily related genes, which could be attributed to the presence of toxic secreted proteins. On the contrary, Chin et al. (2010) [6] demonstrated that expression of xenobiotic metabolism was suppressed in the liver of mice with acute melioidosis. The differences observed may be attributed to the longer duration of exposure to *Burkholderia pseudomallei* and the proteins released during infection in an *in vivo* condition.

In addition, we also observed the acceleration of genes related to glycolysis which may suggest the ability of the host to access high energy during the infection with *B. cepacia*. However, marked up-regulation of glycolysis observed in the ES condition compared to the ML and LBC could be due to the higher secretion of virulence proteins, which may have contributed to the depletion of the host energy release. Significant up-regulation of the biosynthesis of unsaturated fatty acids was also found only in the ES condition, and this may have served to improve the cell structure due to injury caused by the pathogenic *B. cepacia* secretory proteins. When taken together, our data suggest that metabolic pathways were generally activated for the pathogen to survive intracellularly. These metabolic alterations are presumed for the maintenance of host homeostasis, which is beneficial to the host as well as the pathogen.

In this study, down-regulation of the host apoptotic pathway was detected under all the conditions investigated, with the maximum effect observed under the ES condition, which correlates with the findings by Moreilhon and co-workers (2005) [7]. We believe that this could be due to an urge for the host to survive under the ES condition, which was more pathogenic compared to the LBC and ML conditions. Inhibition of apoptosis of the A549 cells would ease replication of the bacteria, prolongs their intracellular survival and favours bacterial persistence.

Activation of the p53 signalling pathway can lead to either the cell cycle arrest or apoptosis [26]. In the present study, the significant down-regulation of p53 was only found in the LBC condition. Interaction of the live *B. cepacia* with the host cells might have caused minimal DNA damage. This was supported by our cell viability test results which showed minimal percentage of cell death. However, an *in vitro* model for the human meninges has provided evidence that secreted proteins from *Neisseria meningitidis* also play a role in the induction of the cell cycle and resistance to apoptosis [27].

Cell cycle progression is a highly complex process which requires coordinated activation of several kinases, some of which are activated upon binding of a cyclin subunit [28]. Our analysis consistently demonstrated that the cell cycle was up-regulated. *GADD45*, through it’s association with *CDC2*, appears to disrupt interaction between *CDC2* and Cyclin B1 and hence induces G2/M arrest. Similar transcriptional profiles were also seen by Zhang et al. (1999) [29], thus suggesting that *B. cepacia* induces G2/M arrest in response to DNA damage to allow time for DNA repair. In addition, activation of the VEGF signaling pathway promotes angiogenesis, which is involved in the normal processes of wound healing. Therefore, exposure of the host to live *B. cepacia* and secretory proteins may have caused injuries or cell damage.

Additionally, cyclooxygenase (COX), an inflammation enzyme involved in release of excessive amounts of *PGE2* (prostaglandin E2) was also highly up-regulated by the host upon exposure to *B. cepacia* secretory proteins, suggesting that *COX* may be an important mediator of *B. cepacia* pathogenesis. The enhanced expression of *COX* leads to further proliferation, reduction in apoptosis and angiogenesis [30,31]. Ejima et al. (2003) [32] suggested that *COX* is an important mediator of the response to bacterial sepsis when they showed that *COX* deficient mice had increased susceptibly to bacterial peritonitis and toxaemia. Thus, in this study, the induction of *COX* upon exposure to secreted products may partially explain the resistance of the epithelial cells to apoptosis.

Many researchers have demonstrated that infections by intracellular pathogens alter the expression of genes encoding pro-inflammatory cytokines and chemokines, which have been implicated as principal mediators during infections of the host in both *in vitro* and *in vivo* systems [6,7,33]. These cytokines and chemokines also function as central mediators in stimulating various host defences systems such as the cytokine-cytokine receptor interactions pathway, signalling pathways and apoptosis and eventually elicit appropriate adaptive immune system. Moreover, C-JUN, in combination with C-FOS, forms the AP-1 early response transcription factor, which up-regulates transcription of a diverse range of genes involved in proliferation and differentiation to defense against invasion and cell damage. In our study, the pro-inflammatory molecules were found to be down-regulated upon infection, and similar evidences have been observed from *in vitro* [33] and *in vivo* [34] studies. However, this is in contrast to the earlier report by Moreilhon and co-workers (2004) [7], which described up-regulation of the interleukins in the human airway cells when in contact with *S. aureus* supernatant.

Our microarray data demonstrated that *B. cepacia* interfered with the transcription of genes involved in the production of pro-inflammatory cytokines. Inhibition of these genes by *B. cepacia* would theoretically result in a reduction of recruitment of the innate immune cells to the site of infection, which in turn could influence the degree of host inflammatory response and bacterial elimination. *B. cepacia* has been shown to modulate the epithelial bactericidal response in favour of its intracellular survival and persistence in the human host, and this process may be associated with disease relapse. Feezor et al. (2002) [35] demonstrated that the release of *TNFα*, *IL-10* and *IL-6* into bactericidal-stimulated whole blood obtained from patients with severe sepsis was significantly decreased compared with the control group. Moreover, Ertel and colleagues (1995) [36] had hypothesised the role of systemic inflammation and an effectively functioning immune system of the host in eliminating invading pathogens. They speculated that during an infection, the pathogens may be made compromised differently depending on the time of exposure. In addition, they have also reported the excessive down-regulation of the synthesis of pro-inflammatory cytokines and their release in the early period after contact with pathogens. This may be an evolutionary process to reduce the incidence of tissue necrosis and the consequential multiple organ failure.

Likewise, Michie et al. (1988) [37] have demonstrated that infusion of high doses of pro-inflammatory cytokines may result in severe host tissue damage, organ failure and death in an *in vivo* condition. We presume that the response of the host cells to *B. cepacia* secretory proteins mimics the physiological action of the host towards toxins. This phenomenon may be referred to as ‘toxin tolerance,’ which is due to a reduced capacity of the cells to synthesise and/or secrete pro-inflammatory cytokines. Granowitz et al. (1993) [38] hypothesised that the phenomenon of toxin tolerance may be due to a defect in transcriptional and/or translational regulation of the host. These indicate that the control of the immune response of the host to toxins that is mediated by *TNFα* and *IL-16* may represent a potent protective mechanism against infection with invasive microorganisms. In this light, the reduced capability of the host cells from septic shock to produce and release adequate amounts of pro-inflammatory cytokines after exposure to toxin may indicate the inability of septic patients to adequately respond to repeated or persisting invasion of microorganisms and to maintain an effective defence system.

In summary, our data is consistent with the interpretation that *B. cepacia* can manipulate A549 cells for its own advantage, allowing prolonged survival inside the harmless environmental niche of the non-phagocytic cells. The data presented clearly indicate that early exposure of the host cells to either live *B. cepacia* or their secretory proteins are similar. Contrary to popular belief, we postulate that *B. cepacia* induced homeostatic responses in the host cell which in turn aid the bacteria to establish persistent infection. Using this knowledge, strategies interfering with essential interactions between *B. cepacia* and the host cell, like apoptosis inhibition, can be exploited in the future to develop an innovative arsenal of therapeutic compounds.

## Supporting Information

Table S1 (DOC)
