# Supplementary material for: Infection of Burkholderia cepacia Induces Homeostatic Responses in the Host for Their Prolonged Survival: The Microarray Perspective
Source: PLoS One. 2013 Oct 7;8(10):e77418. doi: 10.1371/journal.pone.0077418 (PMC3792155; doi:10.1371/journal.pone.0077418)
Supplement: Table S1 — (DOC) [file pone.0077418.s001.doc]

Table S1: Primer sequences of the genes tested

| **Primer** | **Sequence 5’ – 3’** | **Amplicon size (bp)** |
| --- | --- | --- |
| *Β-actin* (F)  *β-actin* (R) | CAC CTT CAC CGT TCC AGT TT  GAT GAG ATT GGC ATG GCT TT | 20  20 |
| *GAPDH* (F)  *GAPDH* (R) | TGT TGC CAT CAA TGA CCC CTT  CTC CAC GAC GTA CTC AGC G | 21  19 |
| *VAM 8* (F)  *VAM 8* (R) | TGG AGG AAA TGA TCG TGT GCG  GTT GCG GAG ATG TTC CAA GTT | 21  21 |
| *RGS2* (F)  *RGS2* (R) | TAC AAG AAG CTA CAA GTG GCT G  CAT GAG GCT CTG TGG TGA TTT | 22  21 |
| *IL-1β* (F)  *IL-1β* (R) | CAC GAT GCA CCT GTA CGA TCA  GTT GCT CCA TAT CCT GTC CCT | 21  21 |
| *NFKB1A* (F)  *NFKB1A* (R) | CTC CGA GAC TTT CGA GGA AAT AC  GCC ATT GTA GTT GGT AGC CTT CA | 23  23 |
| *AKR1B10* (F)  *AKR1B10* (R) | AGA CCC CTT GTG AGG AAA GC  CCC CAG ACT TGA ATC CCT GTG | 20  21 |
| *PADI2* (F)  *PADI2* (R) | GCG TCC CAT AGA CCT CAA AC  CAG AGA ATC GTG CGT GTG TC | 20  20 |
| *TNF* (F)  *TNF* (R) | AGA TGA TCT GAC TGC CTG GG  CAG CCT CTT CTC CTT CCT GA | 20  20 |
| *LTB* (F)  *LTB* (R) | GAC GAA GGA ACA GGC GTT TCT  GTA GCC GAC GAG ACA GTA GAG | 21  21 |

Footnote: F – forward primer; R - reserve primer
